# IsopiRBank: a research resource for tracking piRNA isoforms

**DOI:** 10.1093/database/bay059

**Published:** 2018-06-28

**Authors:** Huan Zhang, Asim Ali, Jianing Gao, Rongjun Ban, Xiaohua Jiang, Yuanwei Zhang, Qinghua Shi

**Affiliations:** Hefei National Laboratory for Physical Sciences at Microscale, The First Affiliated Hospital of USTC, USTC-SJH Joint Center of Human Reproduction and Genetics, The CAS Key Laboratory of Innate Immunity and Chronic Diseases, School of Life Sciences, CAS Center for Excellence in Molecular Cell Science, University of Science and Technology of China, Collaborative Innovation Center of Genetics and Development, Collaborative Innovation Center for Cancer Medicine, Hefei, Anhui 230027, China

## Abstract

PIWI-interacting RNAs (piRNAs) are essential for transcriptional and post-transcriptional regulation of transposons and coding genes in germline. With the development of sequencing technologies, length variations of piRNAs have been identified in several species. However, the extent to which, piRNA isoforms exist, and whether these isoforms are functionally distinct from canonical piRNAs remain uncharacterized. Through data mining from 2154 datasets of small RNA sequencing data from four species (*Homo sapiens*, *Mus musculus*, *Danio rerio and Drosophila melanogaster*), we have identified 8 749 139 piRNA isoforms from 175 454 canonical piRNAs, and classified them on the basis of variations on 5′ or 3′ end via the alignment of isoforms with canonical sequence. We thus established a database named IsopiRBank. Each isoforms has detailed annotation as follows: normalized expression data, classification, spatiotemporal expression data and genome origin. Users can also select interested isoforms for further analysis, including target prediction and Enrichment analysis. Taken together, IsopiRBank is an interactive database that aims to present the first integrated resource of piRNA isoforms, and broaden the research of piRNA biology. IsopiRBank can be accessed at http://mcg.ustc.edu.cn/bsc/isopir/index.html without any registration or log in requirement.

Database URL: http://mcg.ustc.edu.cn/bsc/isopir/index.html

## Introduction

PIWI-interacting RNAs (piRNAs) are small, 26–31 nt single-stranded RNAs that specifically interact with the PIWI proteins, a clade of Argonaute protein family (1). They are mainly expressed in germline, and essential for spermatogenesis (2–5). Many piRNAs are derived from repeat of elements, especially transposable elements (TEs) (6, 7). They can silence the TEs through the cleavage of TE-derived transcripts post-transcriptionally (8–10). piRNAs can also guide PIWI to establish H3K9me3 repressive marks on chromatin at target transposon loci, thereby epigenetically regulate transposons (11, 12). Additionally, piRNAs are reported to act like miRNAs to induce mRNA deadenylation or decay (13–15). piRNAs that were produced from coding genes can also regulate the ‘host’ gene expression (16).

Owing to rapidly increasing high throughput sequencing data, the number of annotated piRNAs in different species and tissues are growing faster than ever (17). piRNAs are found to be quite diverse in number and sequence (18–20). Recently, different length variations of piRNAs have been discovered in several studies (21–23). For example, in Drosophila, piRNAs become shorter with age, accompanied with the derepression of TEs, indicating a strong association between piRNA length variations and biological significance (23). One prominent testicular piRNA, AT-chX-1, has been identified with multiple forms and defined sequences in *Drosophila* (23). Despite progress on piRNA biogenesis, mechanisms that modulate piRNA sequences are still poorly understood. Recently, several 3′–5′ exonucleases have been found to be required for piRNA 3′ end trimming in different species. In *C. elegans*, deficient for PARN-1, a conserved RNase, accumulate untrimmed piRNAs with 3′ extensions and resulted in impaired transcriptome surveillance (24). In silkworms, PNLDC1, an uncharacterized 3′–5′ exonuclease has been identified as Trimmer at piRNA 3′ ends (25). In *Drosophila*, *Nbr*, an established 3′–5′ exoribonuclease, firstly discovered in miRNA isoform (isomiR) biogenesis, is found to be responsible for trimming piRNA 3′ ends and functionally relevant with the repression of TEs (23, 26). Besides, some proteins have been found to modulate piRNA length corporately or antagonistically with exonucleases. BmPAPI, a TUDOR domain-containing protein, can also modulate the length of piRNAs via the cooperative working with PNLDC1 in silkworms (25, 27). Depleting both of them resulted in accumulation of ∼35–40 nt pre-piRNAs that were impaired for target cleavage and are prone to degradation (25). In mouse, HEN methyltransferase 1 (HENMT1) loss-of-function leads to shortened piRNAs at 3′ ends with increased instability, and ultimately male sterility (21). In *Drosophila*, antagonistic roles between Hen1 and Nbr have been found in modulating piRNA 3′ ends (23). These discoveries indicated the existence of piRNA isoforms, like already established known isomiRs. In contrast to well-studied isomiRs, the divergent nature of piRNA biogenesis and sequence, increases the complexity of studies on piRNA isoforms. Until now, several bioinformatics tools, such as piRNABank (28), proTRAC (29), piClust (30), piRNAQuest (31), piRBase (17) and piRNA cluster (32) had been generated for piRNAs and piRNA clusters detection or annotation. However, no bioinformatics resource is yet dedicated for the detection of piRNA isoforms from sequencing data, let alone systematic annotation for piRNA isoforms. We thus established a database named IsopiRBank, to be the first integrative resource that focuses on piRNA isoforms.

**Figure 1.**
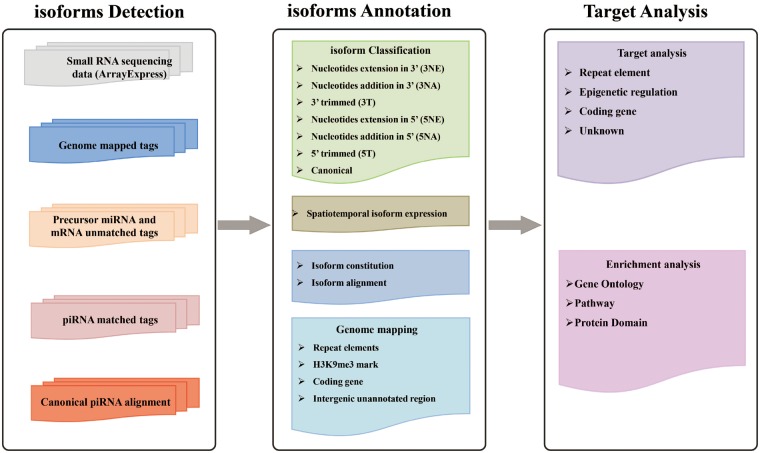
Schematic representation of IsopiRBank Database.

In IsopiRBank, piRNA isoforms are detected through our previously published algorithm CPSS (33), which has been successfully applied to identify piRNAs in human testis (34). In total, 2154 datasets of small RNA sequencing data from four species (*Homo sapiens*, *Mus musculus*, *Danio rerio* and *Drosophila melanogaster*) were analyzed. In view of the fact that a few enzymes responsible for isomiR processing have recently been identified with similar function in modulation of 3′ end of piRNAs, resulting in variations in piRNA length (21, 22, 35), we classified piRNA isoforms with reference to the types of isomiR (36). Specifically, via the alignment of isoforms with canonical sequence, the classification of piRNA isoforms was based on the variations on 5′ or 3′ end, including nucleotides extension, addition or trimming. To be noted that, a specific piRNA isoform can have variations on both ends. In summary, we have identified 8 749 139 piRNA isoforms corresponding to 175 454 canonical piRNAs, and classified them on the basis of variations on 5′ or 3′ end. We thus established a database named IsopiRBank. We organized analytical information for each piRNA isoform, including isoform sequence, expression data, genome origin and metadata associated with each dataset (such as sex, tissue/cell line, developmental stage and genotype) in our database. IsopiRBank also provides target analysis for piRNA isoforms, which is useful to prioritize piRNA isoforms of interest for further functional investigations. Furthermore, enrichment analysis can be done for affected targets. IsopiRBank is implemented in PHP + MySQL + JavaScript+ R and can be accessed at http://mcg.ustc.edu.cn/bsc/isopir/index.html without any registration (Figure 1).

## Materials and methods

### Data collection and pre-processing

In total, processed datasets of 2 154 small RNA sequencing data from four species (*H.sapiens—*1132 datasets, *M.musculus—*457 datasets, *D.rerio—*58 datasets and *D.melanogaster—*507 datasets) were downloaded from ArrayExpress database. Each dataset was manually checked to make sure that they had already been processed with removal of adaptor and filtering out low quality. Metadata associated with each dataset (such as species, tissues, development stage, genotype and sex) was also collected.

### Detection of piRNA isoforms

The data analysis process utilized our previously published algorithm CPSS2.0 (37). Bowtie and BLAST were used for sequence alignment. In the first step, the ‘–n’ mode of Bowtie was used for mapping tags into reference genome (aligned tags had no mismatches in the first ‘10’ bases of the left end). Next, genome mapped tags were matched with precursor miRNA, mature miRNA, canonical piRNA, circRNA, lncRNA, Rfam, repeats and mRNA sequences by using BLAST with default *E*-value (0.01). Considering canonical piRNA length (26–31 nt) and length variations of piRNAs identified in previous studies (21, 24, 25, 38, 39), unmatched tags longer than 14 nt and shorter than 46 nt were matched with canonical piRNA sequences allowing one internal mismatch. These matched tags were considered as piRNA isoforms. Finally, piRNA isoforms were compared with their corresponding canonical piRNAs and variant nucleotides were then extracted.

Precursor miRNA sequences were downloaded from miRBase (40). In our database, canonical piRNA was defined as the piRNAs curated in piRBase (17), piRNABank (28) and NCBI (41), with redundancy removed. The corresponding sequences were downloaded from piRBase (17), piRNABank (28) and NCBI (41) as canonical piRNA sequences. Genome coordinates of each species were updated to the newest version through liftOver tool provided by the UCSC (42).

### Annotation of piRNA isoforms

#### piRNA isoform source

##### Datasets

Accession number in ArrayExpress which the isoform was detected.

##### piRNA isoforms quantification

The expression level of each piRNA isoform detected in a sample was normalized by reads per million (RPM) with the following formula: RPM_*isoform*_ = (*N_isoform_*/*N_all_*) × 10^6^, where *N_isoform_* was the number of reads mapped to the piRNA isoform and *N_all_* was the total number of reads mapped in the sample. To combine all the normalized values among samples, Cumulative RPM and Divided RPM were calculated according to the formula: Cumulative RPM = ∑RPM_*i*_, *i *=* *1*…n*, where RPM_*i*_ is the RPM in sample *i*. Divided RPM = ∑RPM_*i*_/n, *i *=* *1*…n.*

#### Detailed sample information

Sample information is the metadata collected with each dataset, including species, tissues, development stage, genotype and sex, to describe the spatiotemporal expression pattern of piRNA isoforms.

#### piRNA isoforms classification

Since a few enzymes that are previously found to be responsible for isomiR biogenesis, have also been identified with similar function in modulation of piRNA length (21, 22, 35), we classified piRNA isoforms according to their alignment with canonical sequence, and also referred to the rules of isomiR classification (36). piRNA isoforms of piR-hsa-20404 were listed as an example to illustrate the classification (Supplementary Figure S1):

- *Nucleotides extension in 3*′ *(3NE)*: sequence of isoform is longer than the reference sequence at 3′ end, but the extended nucleotides can align with the downstream genome sequence close to piRNA genome loci.
- *Nucleotides addition in 3*′ *(3NA)*: sequence of isoform is longer than the reference sequence at 3′ end, and the added nucleotides cannot align with the downstream genome sequence close to piRNA genome loci.
- *3*′ *trimmed (3TR)*: sequence of isoform is shorter than the reference sequence at 3′ end.
- *Nucleotides extension in 5*′ *(5NE)*: sequence of isoform is longer than the reference sequence at 5′ end, but the extended nucleotides can align with the upstream genome sequence close to piRNA genome loci.
- *Nucleotides addition in 5*′ *(5NA)*: sequence of isoform is longer than the reference sequence at 5′ end, and the added nucleotides cannot align with the upstream genome sequence close to piRNA genome loci.
- *5*′ *trimmed (5TR)*: sequence of isoform is shorter than the reference sequence at 5′ end.

#### Canonical piRNA information

##### Name

piRNA name from piRBase, as well as NCBI accession number are provided for canonical piRNA.

##### Cluster

piRNA isoforms were mapped with piRNA clusters (32). The genome coordinates of the corresponding piRNA cluster are provided.

##### Canonical sequence

Canonical piRNA sequence, with 15 bp of upstream and downstream sequence is provided.

##### Genome mapping

Regulating role of piRNAs is closely associated with the genomic location from where the piRNAs derived (1, 43). Therefore, isopiRBank provided the genome mapping annotation as follows:

(I) *Repeat elements*: piRNAs that matched to repetitive DNA elements annotated by Dfam (44) were defined as repeat elements-derived piRNAs.
(II) *Exon region/coding gene region*: piRNAs that matched to the exon regions or any other coding gene regions (introns, 3′UTR and 5′UTR) were defined as coding gene-derived piRNAs.
(III) *H3K9me3 mark*: piRNAs mapped to H3K9me3 peaks region were predicted to be functional in epigenetic regulation. H3K9me3 peaks were collected form ENCODE (hosted by UCSC) (45).
(IV) *Intergenic unannotated region*: piRNAs that could not match to genome regions mentioned above were defined as intergenic unannotated region-derived piRNAs.

### Target analysis

Potential targets were predicted based on the following two rules:

1. piRNAs antisense to transposons, have function in transposon silencing. piRNA isoforms shorter than canonical piRNAs, are thought to be more instable, accompanied with depression of transposon elements (21, 23, 38).
2. piRNAs, derived from non-transposon intergenic regions, have been found to target diverse mRNAs via a miRNA-like mechanism (13–15, 46). piRNA–mRNA interaction rules were extensively studied in mouse pachytene piRNAs (14, 15, 47). We compared the rules mentioned in both studies in Supplementary Table S1. They both found that piRNAs with mRNA targeting potential had strong biases for U and A residues at nucleotide positions 1 and 10 (15, 47). Therefore, for piRNA isoforms having these sequence characteristics, our database performs target prediction based on the following rules: (i) the minimal continuous base-pairing length is at least 16 nt, starting from position 2 of piRNA. (ii) The maximum mismatches are 3 in a continuous sequence of 20 nt starting from position 2 on piRNAs (15).

Further target analyses for selected piRNA isoforms were also provided. Scatter plot was used to illustrate the differences in piRNA: mRNA interactions, in which isoforms-affected targets were supposed to deviate from the diagonal. Finally, the enrichment analysis was carried out for piRNA isoform affected targets. The Gene Ontology, Pathway and Protein Domain information used for enrichment analysis were retrieved from DAVID (48). The enrichment *P-*values were quantitatively measured by Fisher’s exact test and Bonferroni correction was calculated when an adjustment was made to *P-*values.

### Database accession and web interface

**Figure 2.**
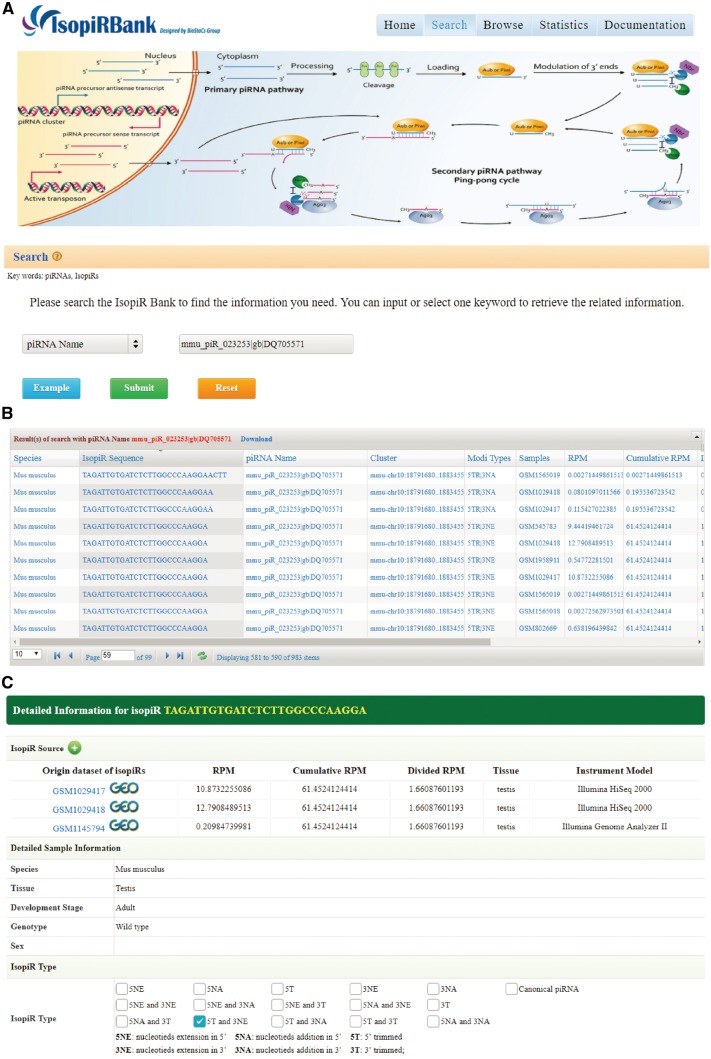
Screen shots of User Interface—Search. One of the three provided options, piRNA name, was used to search the database (**A**). Upon clicking the submit button, the search results will be displayed in tabular form (**B**). Extensive information of the isoform (**C**).

The IsopiRBank database is accessible, without any login requirements, via the URL http://mcg.ustc.edu.cn/bsc/isopir/. It includes a search engine to find the piRNA isoforms of interest. Users have three searching options (Figure 2A): (i) They can search a piRNA isoform sequence with further options to select the matching rules for input sequence (equal, start with, end with or contain); (ii) they can search for all piRNA isoforms of a given piRNA by using either piRNABank ID or piRBase ID; (iii) they can search for a repertoire of piRNA isoforms expressed in a specific tissue. The search results will be formatted and displayed in a tabular form (Figure 2B). Users can click the piRNA isoform sequence within the table to view more detail information on a new page along with its canonical piRNA information. This new page provides extensive information of piRNA isoform, including sample source (from which sample the isoform is detected), expression information (RPM, Cumulative RPM, Divided RPM), and other information from metadata (tissue/cell line, development stage, genotype and sex of the corresponding sample) (Figure 2C).

**Figure 3.**
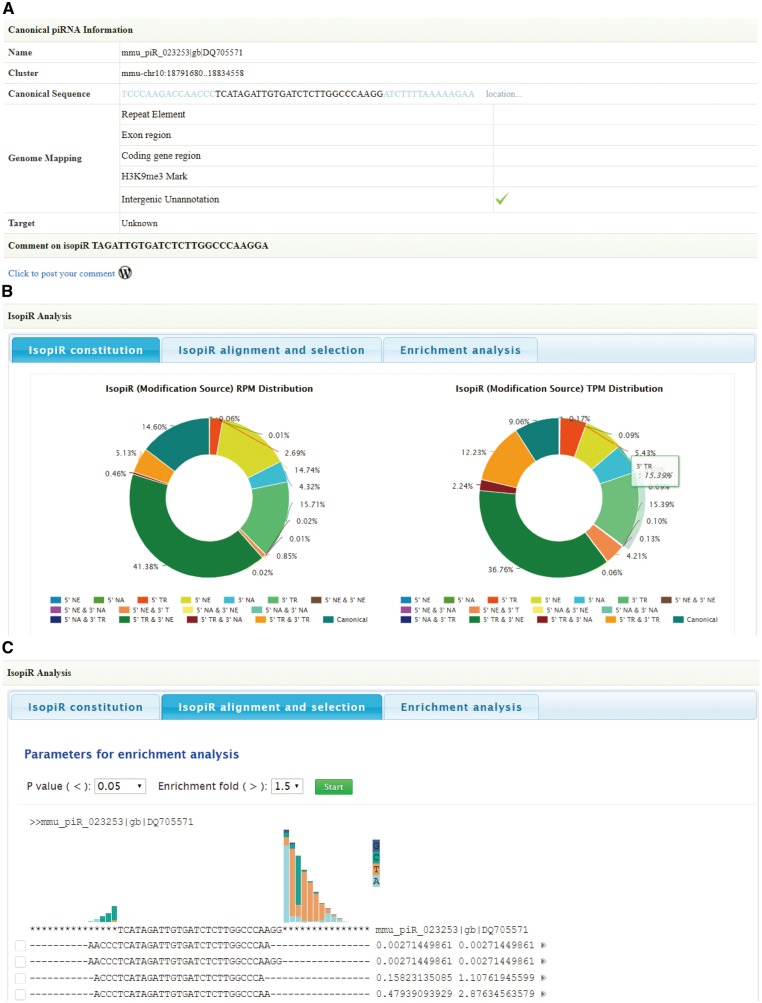
Screen shots of User Interface—Annotation. Information of canonical piRNAs (**A**). Diagrams to illustrate piRNA isoforms constitution (**B**). Select piRNA isoforms for target analysis (**C**).

**Figure 4.**
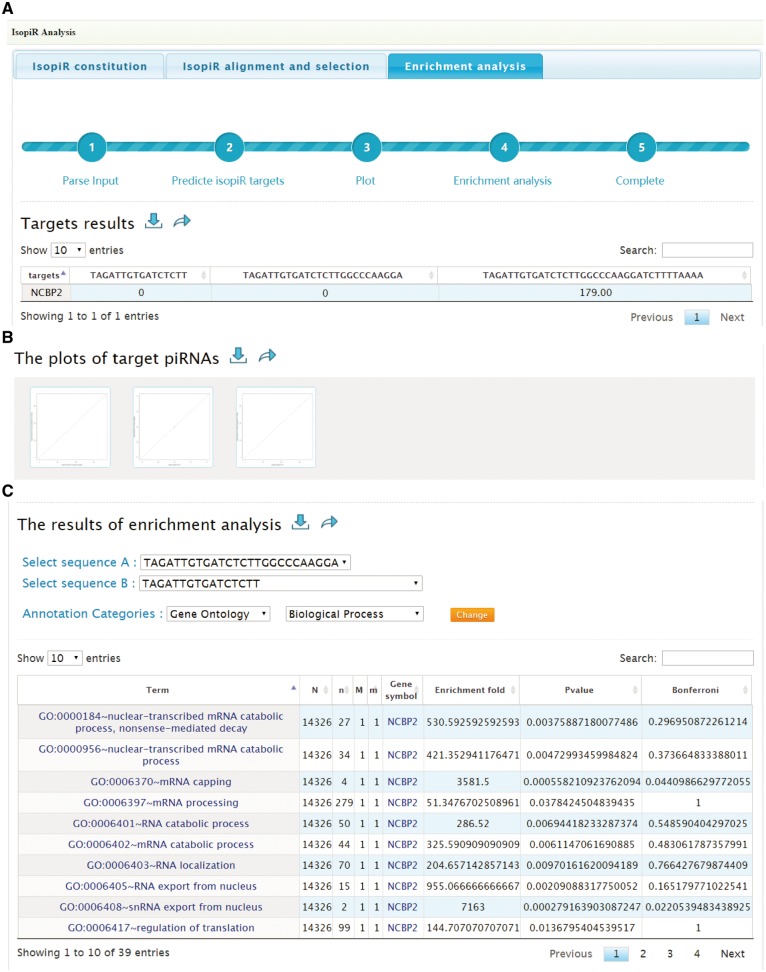
Screen shots of User Interface—Enrichment analysis. Target list (**A**) and scatterplots (**B**) to illustrate the effects of selected piRNA isoforms on targets selection. Enrichment results of target genes (**C**).

For a better understanding of the genome origin and potential function of piRNA isoforms, information of canonical piRNAs was also provided below, including chromosome location, canonical sequence, genome mapping results and target (as described in the ‘Genome mapping annotation’ and ‘Target analysis’ sections) (Figure 3A). Furthermore, piRNA isoform analysis module is designed to integrate analysis of all piRNA isoforms from a canonical piRNA. Information will be displayed in three tabs:

- *Isoform constitution*: In this tab, diagrams are provided to illustrate the percentage of each piRNA isoform type based on their expression (Figure 3B).
- *Isoform alignment and selection*: In this tab, all the piRNA isoforms are aligned with the canonical sequence. For each isoform, RPM and Cumulative RPM values are displayed next to its sequence. Users can click the arrowhead button for more annotation information, including isoform types and tissue information. The query isoform sequence is highlighted in red. Users can select piRNA isoforms of their interest (range from 2 to 6) for target analysis (Figure 3C). By clicking on the ‘start’ button, target analysis will start.
- *Enrichment analysis*: When the analysis is finished, this tab will provide the targets list (Figure 4A) and scatterplots (Figure 4B) to illustrate the effects of selected piRNA isoforms on targets selection. Moreover, functional enrichment analysis for these affected targets is also available to users. By selecting the piRNA isoforms’ sequences (two piRNA isoforms for one analysis), Annotation Categories (Gene Ontology, Pathway, Protein Domains) and subsets of each category from the drop-down box, users can also overview the effect of isopiR-affected targets on downstream biological processes, pathways or protein domains. The enrichment fold, *P-*values (Fisher’s exact test) and Bonferroni adjusted *P-*values can be optimized to refine the results (Figure 4C). Results can be saved by clicking download button next to them.

### piRNA isoforms overview and discussion

We detected 8 749 139 piRNA isoforms from 175 454 canonical piRNAs in four species (Supplementary Table S2). Around half piRNA isoforms were detected in two or more datasets, thus providing a reliable evidence for their existence (Supplementary Figure S2). The statistical results of piRNA isoforms with different types were listed in Supplementary Figure S3A. The most abundant piRNA isoforms are those with length variations at 3′ end (3NA, 3NE and 3T) in all four species (Supplementary Figure S3B). This is consistent with that modification of 3′ end within various RNA molecules has been validated as a general and conserved process, which plays an important role in determining RNA biological fate (49). Nucleotides addition or trimming of 3′ ends are major mechanisms that turnover small RNAs, such as miRNA, siRNA and piRNA (26, 38, 50, 51). These modifications found in miRNA 3′ ends occurred enzymatically through the activity of nucleotidyl-transferases or exoribonuclease, such as PAPD4, PAPD5, ZCCHC11, TUT1 and *Nbr* (35, 52). *Nbr* has been demonstrated to function in 3′ trimming of piRNAs (21, 22). Beside this, we also found a small fraction of piRNA isoforms with 3′ or 5′ NE (Supplementary Figure S3B), which are similar to isomiRs that resulted from variations in the Drosha and Dicer cleavage sites within pre-miRNA (53). Although no secondary structure is found in piRNA processing, long piRNA precursors are reported to be cleaved by an endonuclease, possibly Zucchini, during primary piRNA biogenesis (54). In secondary piRNA biogenesis, AGO3 and Aub act in a complementary fashion to cleave sense and antisense transposon transcripts via their Slicer activities (43). Therefore, piRNA isoforms with 3′ or 5′ NE may be generated during the cleavage steps in primary and secondary piRNA biogenesis, by means of unknown imperfect cleavage mechanism.

We have also analyzed the number of piRNA isoforms shared between any two species in the database. Species having closer genetic/evolutionary relationship shared more piRNA isoforms (Supplementary Table S3, Supplementary Figure S4). This is inconsistent with their canonical piRNAs that are poorly conserved (18), suggesting that these types of piRNA isoforms may have biological importance during evolutionary process and may be involved in the regulation of same biological processes across different species.

IsopiRBank also provides canonical piRNA genome mapping annotation to illustrate the downstream function change of corresponding piRNA isoforms. It has been demonstrated that piRNAs are undergoing 3′ end trimming during aging, coupled with derepression of TEs (23). Therefore, piRNA isoforms with 3′ trimming may have impaired TE repression. Besides, piRNA isoforms with 3′ NA may also lead to derepression of downstream TE, due to their decreased stability. However, it is not yet known that how and to what extent the repressive role of these piRNA isoforms is influenced. For piRNAs mapped to intergenic unannotated region or coding gene regions, they may post-transcriptionally silence target mRNA, like miRNA machinery. For example, mouse pachytene piRNAs instruct massive mRNA elimination during late spermiogenesis, and >45 075 piRNA–mRNA pairs have been predicted in mouse elongating spermatids (14). In order to explore whether piRNA isoforms are involved in this process, we analyzed piRNA isoforms from small RNA sequencing data of pachytene spermatocyte and round spermatid, both of which expressed pachytene piRNAs (55). We found that the piRNA isoforms were much more abundant than canonical piRNAs (Supplementary Figure S5A), and most piRNA isoforms were mapped to the coding gene region and H3K9me3 mark (Supplementary Figure S5B). These piRNA isoforms with diversified targeting capacity, together with canonical piRNAs, made the real situation that how pachytene piRNAs regulate mRNAs much more sophisticated than previously thought.

### Availability and future directions

The number of piRNAs is increasing rapidly along with their piRNA isoforms. We will therefore update IsopiRBank and integrate more information supporting piRNA isoforms functional analysis routinely. In the future, we will also integrate piRNA isoforms from other species with available small RNA sequencing data and focus on analysis of downstream change of piRNA isoforms, especially when their targets are non-coding elements.

## Conclusion

In conclusion, we constructed the first piRNA isoforms database, isopiRBank. Our database stores piRNA isoforms detected from small RNA sequencing data, and makes detailed classification and integrates annotation information as well as genome mapping results. The database also provides convenient search results for piRNA isoforms of users’ interest. Furthermore, target analysis and enrichment analysis will help users to reveal piRNA isoforms’ roles in certain biological process.

## Supplementary data

Supplementary data are available at *Database* Online.

## Supplementary Material

Supplementary Data
