# Supplementary material for: IsopiRBank: a research resource for tracking piRNA isoforms
Source: Database (Oxford). 2018 Jun 28;2018:bay059. doi: 10.1093/database/bay059 (PMC6025188; doi:10.1093/database/bay059)
Supplement: Supplementary Data [file bay059_supp.zip › Supplementary Figures.docx]

Supplementary Figure S1.


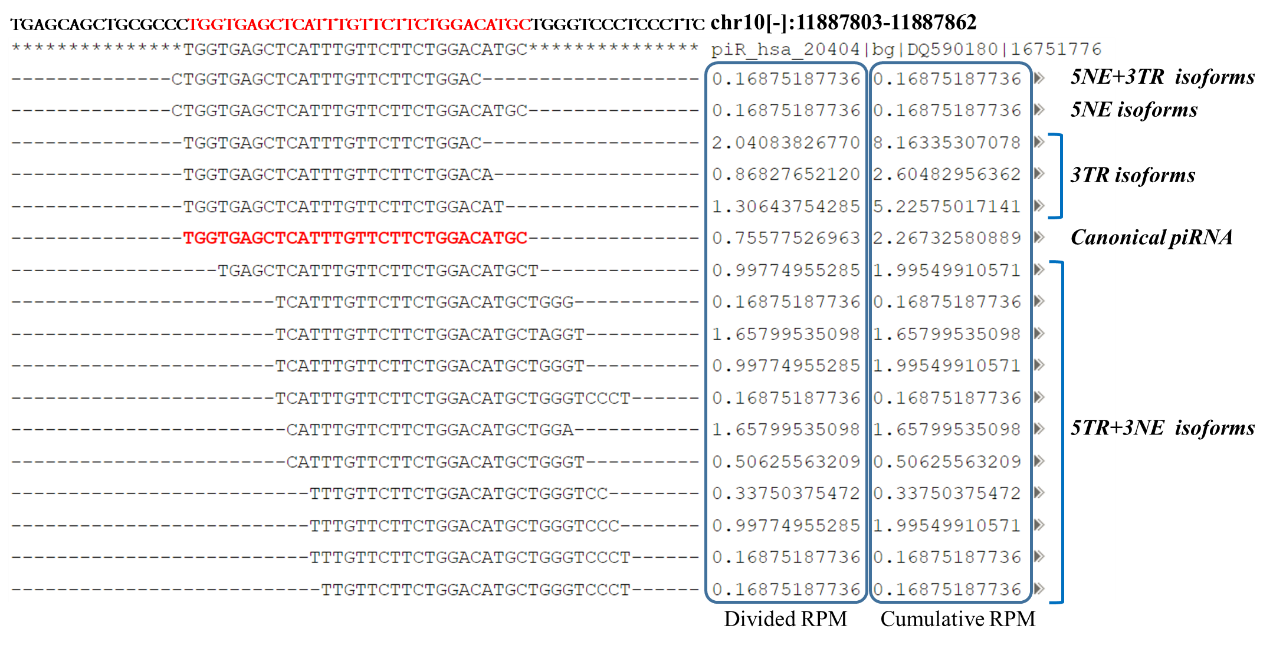


Supplementary Figure S1. An example to illustrate the different types of piRNA isoforms from piR_hsa_20404|DQ590180. These isoforms were detected from small RNA sequencing data from human fetal ovaries (1).

5NE: Nucleotides extension at 5’ end; 5TR: Nucleotides extension at 5’ end

3NE: Nucleotides extension at 3’ end; 3TR: Nucleotides trimmed at 3’ end

Supplementary Figure S2.


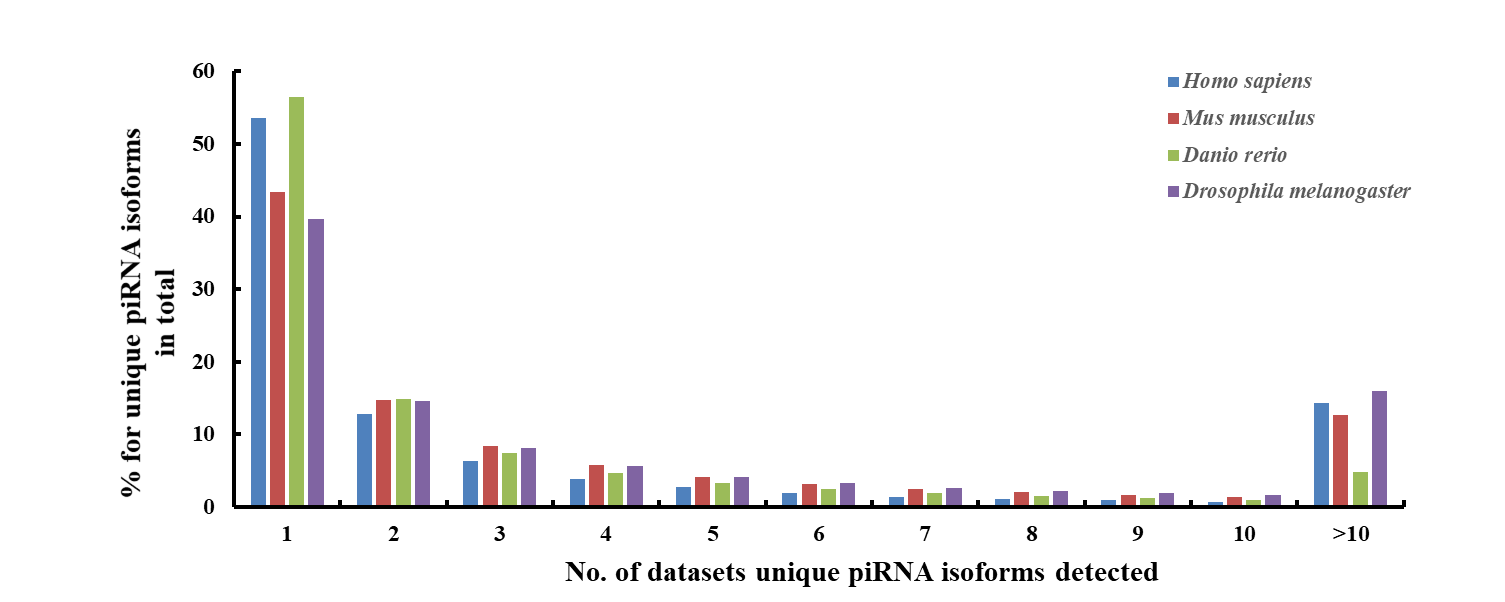


Supplementary Figure S2. The proportion of unique piRNA isoforms that can be detected in one or more datasets.

Supplementary Figure S3

**A**


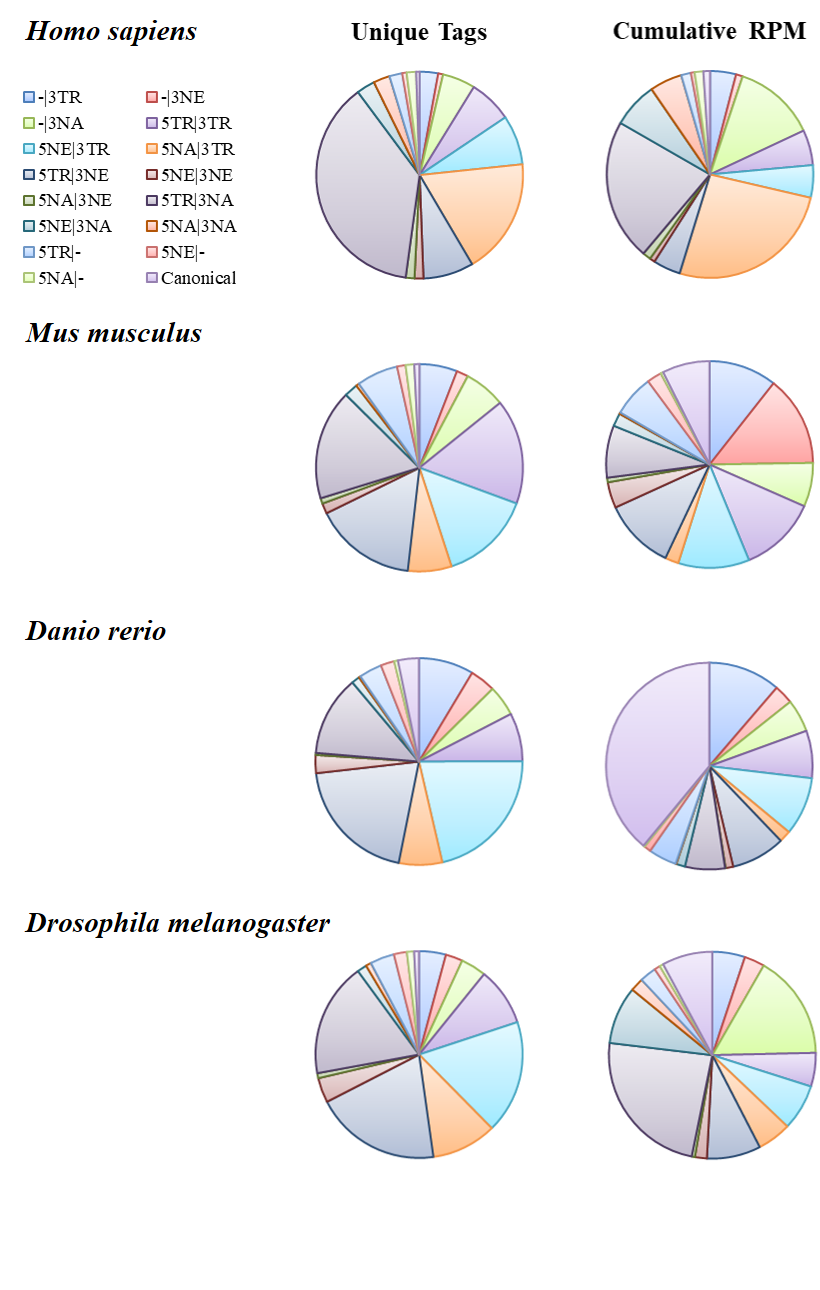


**B**


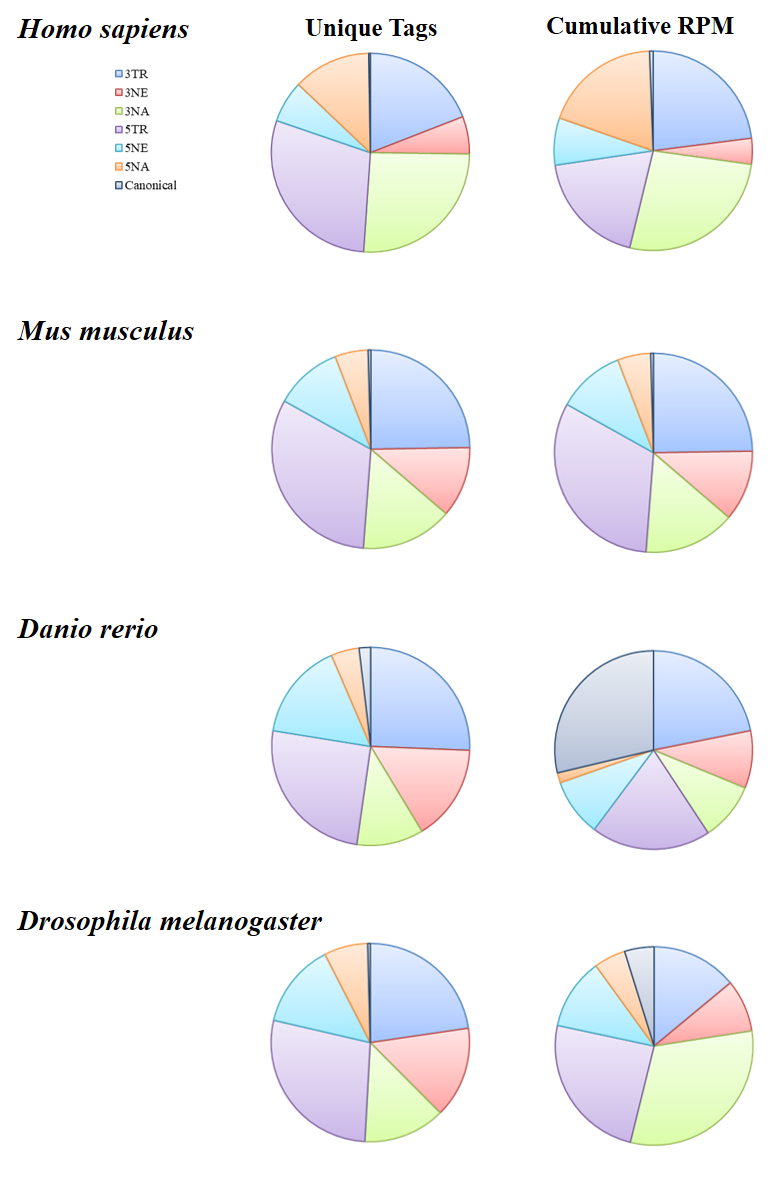


Supplementary Figure S3. (A) The statistical results of different piRNA isoform types on unique tags and cumulative RPM, in 4 species. (B) The statistical results of piRNA isoforms with 3’ or 5’ end modifications on unique tags and cumulative RPM, in 4 species.

Supplementary Figure S4


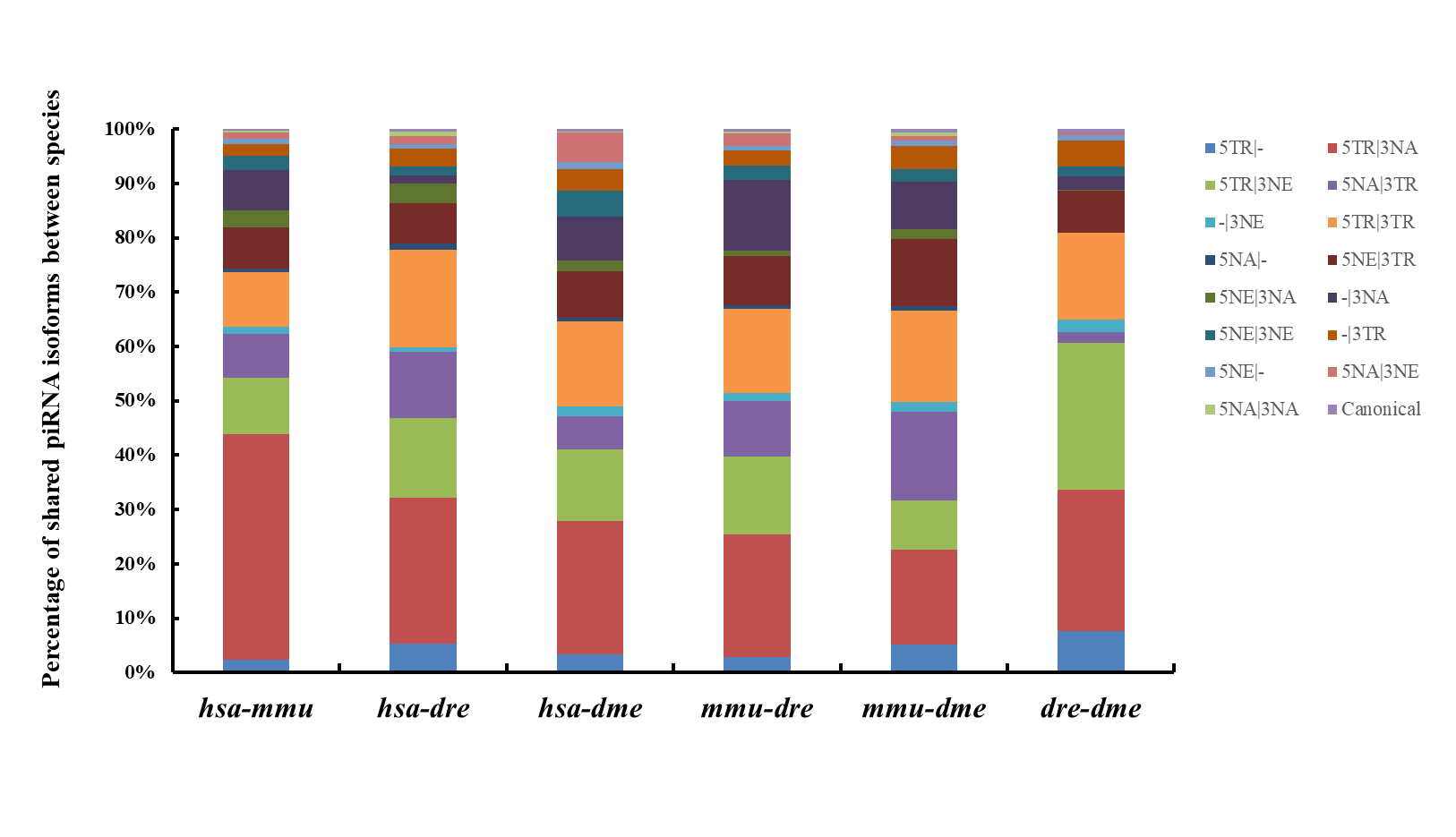


Supplementary Figure S4. The ratio of different isoform types in all piRNA isoforms shared between any two species.

Supplementary Figure S5

**A**


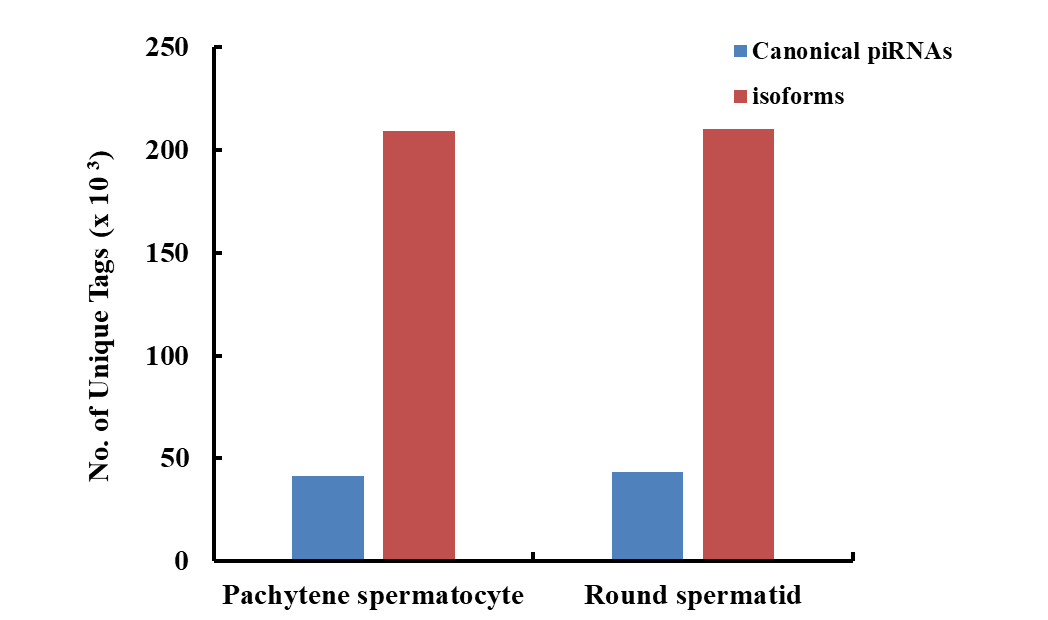


**B**


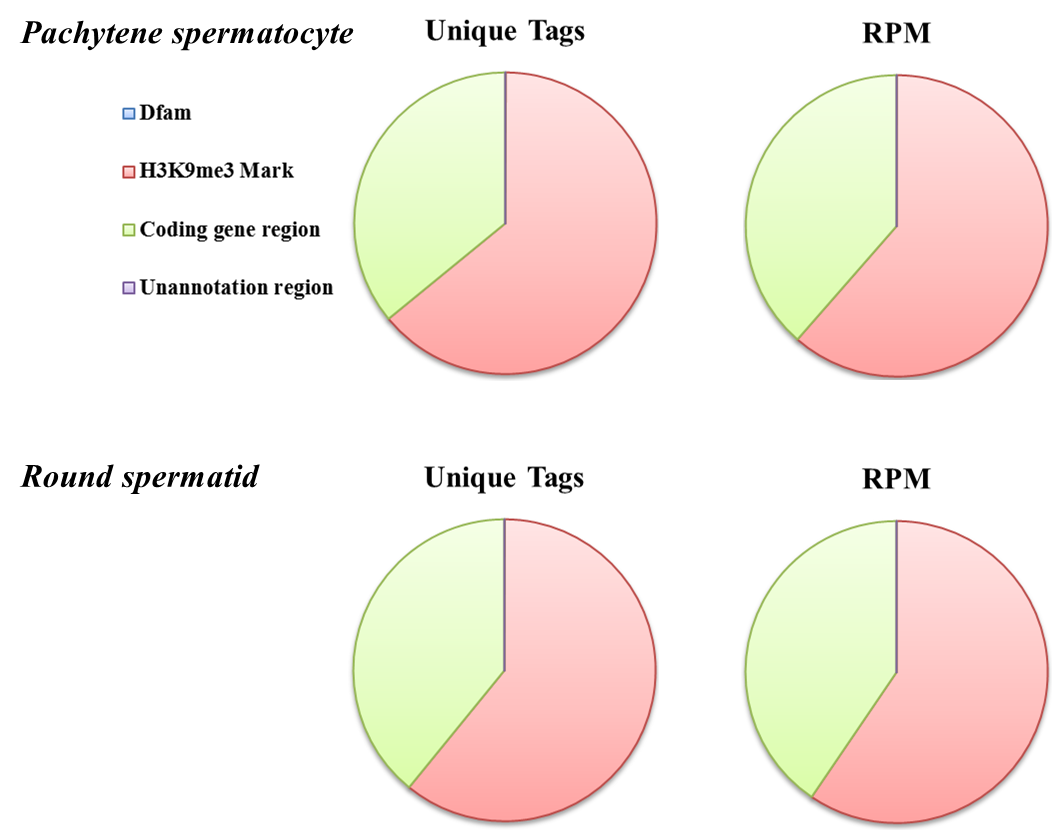


Supplementary Figure S5. (A) The statistical results of canonical piRNAs and piRNA isoforms detected in mouse pachytene spermatocyte and round spermatid, respectively. (B) The genome mapping results of piRNA isoforms detected in mouse pachytene spermatocyte and round spermatid.

Reference:

1. Roovers, E.F., Rosenkranz, D., Mahdipour, M., Han, C.T., He, N.N., Lopes, S.M.C.D., van der Westerlaken, L.A.J., Zischler, H., Butter, F., Roelen, B.A.J. *et al.* (2015) Piwi Proteins and piRNAs in Mammalian Oocytes and Early Embryos. *Cell Rep*, **10**, 2069-2082.
