# Supplementary material for: IsopiRBank: a research resource for tracking piRNA isoforms
Source: Database (Oxford). 2018 Jun 28;2018:bay059. doi: 10.1093/database/bay059 (PMC6025188; doi:10.1093/database/bay059)
Supplement: Supplementary Data [file bay059_supp.zip › Supplementary Tables.docx]

| Supplementary Table S1 Comparison of reported piRNA-mRNA interaction rules | | |
| --- | --- | --- |
| Studies | Continuous base-pairing length | Maximum of mismatch tolerated |
| Goh, Falciatori et al. 2015 (1) | Perfect matching in Pos.2-11(Primary seed) | 4 mismatches in Pos. 12–21 (Secondary seed) |
| Zhang, Kang et al. 2015 (2) | Continuous matching in Pos.2-17 at least | 3 mismatches in Pos.2-21 |

| Supplementary Table S2. Data statistics in IsopiRBank | | | | |
| --- | --- | --- | --- | --- |
| Species | No. of datasets | No. of total canonical piRNAs | No. of canonical piRNAs with isoforms | No. of piRNA isoforms |
| *Homo sapiens* | 1,132 | 32,826 | 12,571 | 214,517 |
| *Mus musculus* | 457 | 74,722 | 68,876 | 4,401,468 |
| *Danio rerio* | 58 | 82,786 | 73,160 | 1,798,621 |
| *Drosophila melanogaster* | 507 | 22,336 | 20,847 | 2,334,533 |

| Supplementary Table S3. Number of shared piRNA isoforms between species | | | | |
| --- | --- | --- | --- | --- |
|  | *Homo sapiens* | *Mus musculus* | *Danio rerio* | *Drosophila melanogaster* |
| *Homo sapiens* | - | 10,639 | 1,850 | 1,290 |
| *Mus musculus* | 10,639 | - | 4,954 | 1,060 |
| *Danio rerio* | 1,850 | 4,954 | - | 282 |
| *Drosophila melanogaster* | 1,290 | 1,060 | 282 | - |

References:

1. Goh, W.S.S., Falciatori, I., Tam, O.H., Burgess, R., Meikar, O., Kotaja, N., Hammell, M. and Hannon, G.J. (2015) piRNA-directed cleavage of meiotic transcripts regulates spermatogenesis. *Gene Dev*, **29**, 1032-1044.

2. Zhang, P., Kang, J.Y., Gou, L.T., Wang, J.J., Xue, Y.C., Skogerboe, G., Dai, P., Huang, D.W., Chen, R.S., Fu, X.D. *et al.* (2015) MIWI and piRNA-mediated cleavage of messenger RNAs in mouse testes. *Cell Res*, **25**, 193-207.
